# Supplementary material for: The Complete Chloroplast Genome and the Phylogenetic Analysis of Fimbristylis littoralis (Cyperaceae) Collected in Cherry Blossom Nursery
Source: Int J Mol Sci. 2025 Mar 5;26(5):2321. doi: 10.3390/ijms26052321 (PMC11901024; doi:10.3390/ijms26052321)
Supplement: Supplementary file 1 [file ijms-26-02321-s001.zip › Figure S1.pdf]

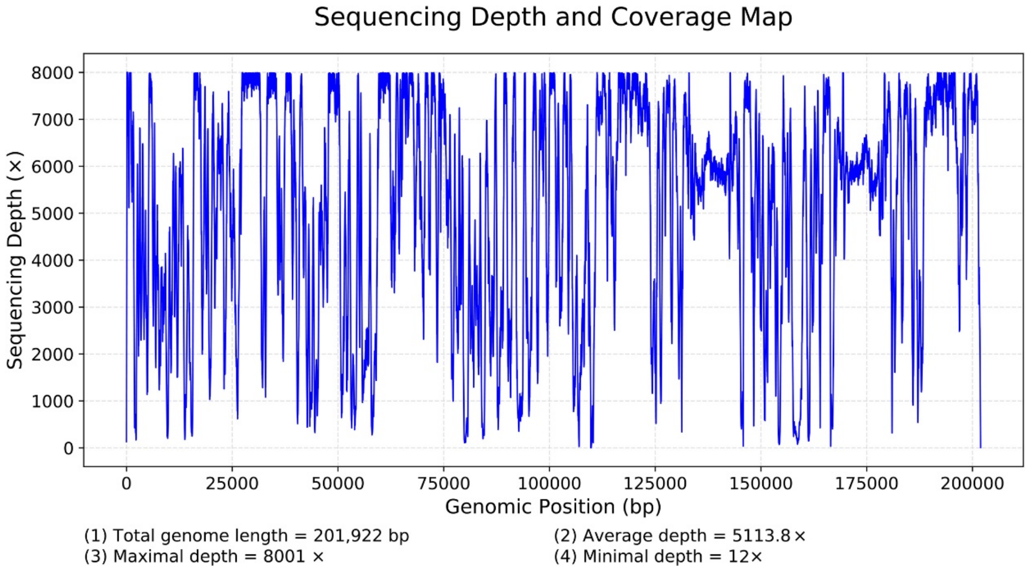

Supplementary Figure S1. Sequencing depth of the chloroplast genome of *Fimbristylis littoralis*.
